# Shared Makeup Cosmetics as a Route of *Demodex folliculorum* Infections

**DOI:** 10.1007/s11686-020-00332-w

**Published:** 2021-01-19

**Authors:** Aleksandra Sędzikowska, Katarzyna Bartosik, Renata Przydatek-Tyrajska, Monika Dybicz

**Affiliations:** 1 Department of General Biology and Parasitology, Medical University of Warsaw, Chałubińskiego 5, 02-004 Warszawa, Poland; 2 Chair and Department of Biology and Parasitology, Medical University of Lublin, Radziwiłłowska 11, 20-080 Lublin, Poland; 3 Reno-Med Non-Public Health Care, Podkowy 87, 04-937 Warszawa, Poland

**Keywords:** Demodicosis, *Demodex folliculorum*, *Demodex* transmission, Transmission via cosmetics, Blepharitis

## Abstract

**Purpose:**

The aim of the study was to examine *Demodex* survival in makeup cosmetics, i.e., powder cream, mascara, and lipstick, and to determine whether cosmetics shared with others can be a source of *D. folliculorum* infection.

**Methods:**

Live *D. folliculorum* adults were placed in cosmetic samples and their motility was observed under a microscope. The mites were fully or partially immersed in the powder cream and lipstick, and only partially immersed in the mascara. Partial immersion means that only the opisthosoma was covered by the cosmetic, whereas the gnathosoma and podosoma had no contact with the cosmetic. Cessation of motility was regarded as a sign of death.

**Results:**

In the control (mites placed on a microscope slide with no cosmetics), the survival time was 41.2 h. *D. folliculorum* that were immersed fully or partially in the lipstick substrate were viable for 38.5 h and 148 h, respectively. The survival time of the mites at full and partial immersion in the powder cream was 0.78 h and 2.16 h, respectively. The average survival time in the mascara was 21 h.

**Conclusions:**

Makeup cosmetics used by different individuals at short intervals (from several hours to several days) can be a source of transmission of *Demodex* sp. mites.

**Supplementary Information:**

The online version contains supplementary material available at 10.1007/s11686-020-00332-w.

## Key Points

**Table:** 

This study investigates the relationship between sharing cosmetics and *Demodex folliculorum* infection.
The results show that mites can survive from few hours to few days in makeup cosmetics depending on its type and composition.
Clients in drugstores using makeup cosmetic testers are at risk of *Demodex folliculorum* transmission via this route.

## Introduction

*Demodex* mites are permanent residents of pilosebacious units in humans and other mammalian species. Two species, i.e., *Demodex folliculorum and Demodex brevis*, have been found to infest humans. The predilection sites mainly include the face, scalp, and chest, but the mites can also colonise other parts of the body [1, 2]. *D. folliculorum* is most often present in hair follicles, whereas *D. brevis* attacks sebaceous glands. The infestation can be both symptomatic and asymptomatic. Such ocular symptoms as itching, redness of eyelids, and lacrimation are reported most frequently [3]. Ocular demodicosis is also considered as a risk factor of recurrence of pterygium [4]. In some patients, the presence of these mites may be associated with blepharitis, rosacea, chalazion, perioral dermatitis, or idiopathic follicular mucinosis of the head and neck [5–10]. Cases of local dermatological lesions caused by increasing *Demodex* populations have been reported as well [11].

In the case of mites that are permanent human parasites, such as *Demodex* spp. or *Sarcoptes scabiei*, it is believed that the invasion requires human skin contact with live mites directly or via an indirect route.]. 241 sequences from the mitochondrial genome of *D. folliculorum* were analyzed and the haplotypes were much more likely to be shared within families than between unrelated individuals. Molecular analyses indicate that frequent close physical contact leads to mite transmission [12]. Direct contact or eggs present in dust as well as contact with infected towels, blankets, or sponges are the possible routes of *Demodex* spp. infection described in the literature [13, 14]. The use of facial creams or eyeliners has been considered as a potential route of *Demodex* spp. infections; however, no studies have addressed the issue of the length of survival of these mites in cosmetics.

Cosmetics are shared not only in households. Before purchase, cosmetic testers available in drugstores can be used or makeup can be done as a special offer by a beautician, who uses the same cosmetics in many customers. Whether such consumer behaviour can become a potential route of *Demodex* spp. infection is unknown. Therefore, the aim of the study was to determine the length of survival of *Demodex* mites in commonly used makeup cosmetics, i.e., powder cream (fluid foundation), mascara, and lipstick, and to find out whether shared cosmetics could be a source of *D. folliculorum* infection.

## Materials and Methods

### Research Procedures

**Fig. 1:**
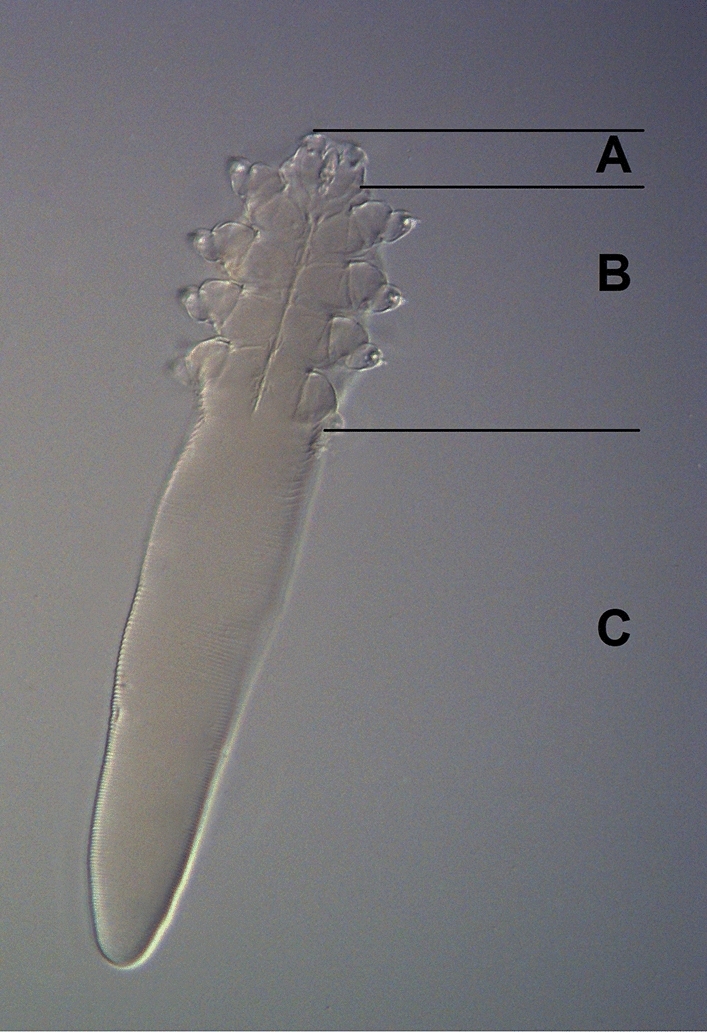
Morphological elements of *Demodex folliculorum*: A – gnathosoma, B – podosoma, C – opisthosoma (original magnification 200x)

Live *Demodex* mites collected from patients reported for consultation with an aesthetic medicine doctor in Warsaw, Poland. The investigation procedures involving the volunteer patients in the study were approved by the local Bioethics Committee at the Medical University of Lublin (approval no. KE-0254/122/2018). Individual patients' consent to participate in the study was obtained as well. The mites were collected from the patients with a method of lash sampling with sterile tweezers. Eyelashes from the left and right eye were sampled for the study. The eyelashes were placed directly on the microscope slide. A Zeiss Primo Star light microscope (magnification from 40 × to 400 × ) was used to detect the presence of *Demodex* spp. in the collected material. The presence of *Demodex* spp. adults, juvenile forms, or eggs on the slide indicated that the sample was positive. Further analyses were performed only on positive slides with the presence of adults (Fig. 1), as their legs are better developed than in juvenile stages and the movement of their legs is more clearly visible. All adult forms were observed under a microscope every 5 min and their continuous motility was recorded. The analyses of mite viability in the cosmetics were carried out only on specimens that moved all the legs and gnathosoma. Mites showing minimal movement (e.g., only the gnathosoma or one pair of legs) were not qualified for further tests. Samples marked as positive were used in the study on the day of sampling.

The survival of *Demodex* mites was examined in basic makeup cosmetics, i.e., mascara, lipstick, and powder cream. Each cosmetic was placed on a microscope slide. Next, the mites were gently transferred directly onto the tested cosmetic and no coverslip was used. A single mite was placed on each microscopic slide. *Demodex* mites transferred to a slide with the cosmetic did not change the place but only moved their legs and gnathosoma and stayed on the slide. Colourless lipstick was used to facilitate the observation of the mites. *Demodex* could be on the surface of the cosmetic or deeper that is why they were immersed either fully or partially in the powder cream and lipstick. Partial immersion means that only the opisthosoma (fragment C marked in Fig. 1) was covered by the cosmetic, whereas the gnathosoma and podosoma (fragments A and B marked in Fig. 1) had no contact with the cosmetic. Specimens were transferred onto the mascara without full immersion, as its dark colour would hamper observations of these small mites. *Demodex* mites placed on the microscope slide with no cosmetics were the control sample. No coverslips were placed on the slides with the mites. All samples were transferred to a humid chamber to prevent desiccation. The slides were stored at room temperature of 22 °C and 60% RH to mimic drugstore and home storage conditions.

The *Demodex* spp. specimens were observed under a light microscope at various intervals. Their viability was assessed according to the following criteria:

- (+ + +) high viability (the mite moved the legs and gnathosoma; sometimes, the entire body moved)
- (+ +) moderate viability (the mite moved the legs and gnathosoma but at a definitely slower pace)
- (+) low viability (the mite exhibited minimal movements of the claws only or gently moved the gnathosoma)
- (−) no motility (the mite did not move any part of the body during the observation).

Mites assigned to categories (+), (+ +), and (+ + +) were considered alive. Each specimen that showed no motility was observed three more times at a several-minute interval. When the subsequent observations revealed no signs of life, the specimen was regarded dead and the first time point of cessation of movement was recorded. The frequency of observations depended on the motility of the mites. In the case of the very high viability (+ + +), the observations were conducted every 2–4 h. When the viability declined with time (+), the observations were conducted more frequently (every 5–10 min) to record the time point of cessation of movement. *Demodex* mites with low viability (+) were observed at high magnification (400 ×) to notice even the slightest movements indicating that the specimen was still alive. Sometimes, only a claw or a part of the gnathosoma was found to move. In the case of an immotile specimen, the exact time of cessation of movement was recorded, and the mite was checked three times to monitor any signs of life. When there was no movement in each subsequent observation, the specimen was assumed to have died at the first time point recorded.

Commonly available facial cosmetics, i.e., powder cream, mascara, and lipstick, were selected for the study. The chemical composition of the cosmetics including the first six ingredients with the highest concentration was as follows:

Powder cream: dimethicone, water, dipropylene glycol, alcohol denat., nylon-12, butylene glycol.

Lipstick: cera microcristallina, octyldodecanol, hydrogenated polydecene, Ricinus communis seed oil, cetyl palmitate, ethylhexyl methoxycinnamate,

Mascara: water, Copernicia cerifera cera, cera alba, glyceryl stearate, Euphorbia cerifera cera, stearic acid.

### Statistical Analysis

The following tests were used to check the difference in the survival of the mites in the different cosmetics:

- Student’s *t* test–comparison of the survival of the mites in two groups (normality of distribution and uniformity of variance),
- Cochran-Cox test–comparison of the survival of the mites in two groups (normality of distribution but no homogeneity of variance),
- Mann–Whitney *U* test–comparison of the survival of the mites in two groups (no normality of distribution),
- Kruskal–Wallis ANOVA test–comparison of the survival of the mites in six groups (no normality of distribution and no homogeneity of variance). The Kruskal–Wallis multiple comparison (post-hoc) test was used to analyse the significance of the differences in the survival between the substrates.

A *p* value of < 0.05 was considered statistically significant. Statistical calculations were carried out using the STATISTICA 10 PL statistical package.

## Results

From 10 to 18 *D. folliculorum* adults, i.e., the species present in the examined patients, were subjected to observations in each cosmetic substrate. In total, the survival of the mites was examined in 77 samples (28 samples of the lipstick, 25 samples of the powder cream, 14 samples of the mascara, and 10 control samples). The in vitro survival time of the *Demodex* mites in the selected cosmetics and in the control sample is presented in Table 1.

**Table 1:** Overall survival of *Demodex folliculorum* in the cosmetics and control group (descriptive statistics)

Substrate	*n*	Survival time (h)					*p*
		Mean	SD	Median	Min	Max	
Control	10	41.2	22.2	42.0	9.0	84.0	0.7269^(1)^
Lipstick–full immersion	18	38.5	17.7	36.5	7.0	69.0	
Lipstick–partial immersion	10	148.1	63.1	147.0	67.0	260.0	
Powder cream–full immersion	15	0.79	0.64	0.52	0.25	2.30	0.0000^(3)^
Powder cream–partial immersion	10	2.17	1.11	1.86	0.88	4.53	0.0004^(2)^
Mascara–partial immersion	14	21.4	14.8	19.0	2.5	56.0	0.0154^(1)^

*SD* standard deviation, *n* number of samples

*p* < 0.05, (1) Student’s *t* test, (2) Cochran-Cox test, (3) Mann–Whitney *U* test

**Fig. 2:**
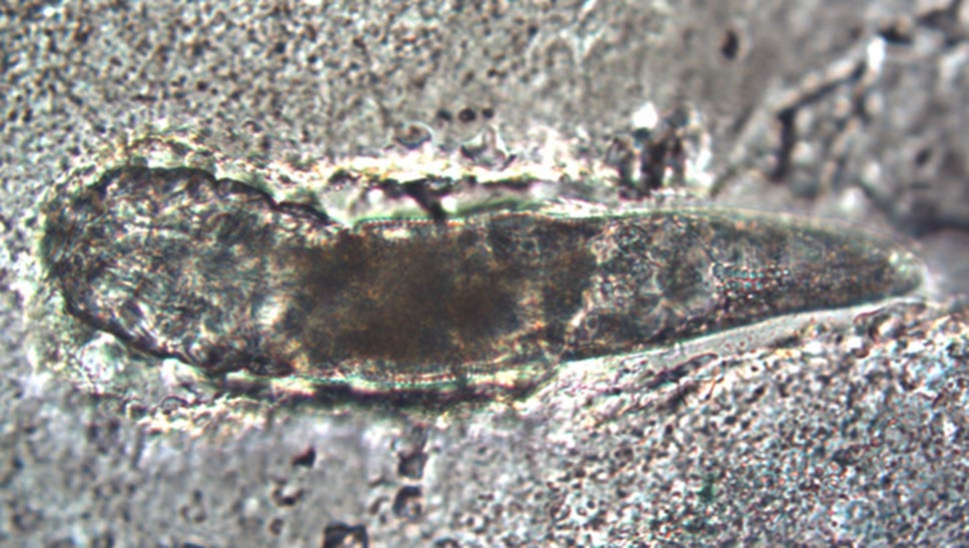
Adult *Demodex folliculorum* fully immersed in colourless lipstick (original magnification 200x)

The survival time was the longest in the case of *D. folliculorum* immersed in the lipstick- 69 h and 260 h for the fully and partially immersed mites, respectively (Fig. 2). In the mascara, the mites were able to stay alive for as long as 56 h. In the powder cream samples, the overall survival time of *D. folliculorum* was the shortest, i.e., maximum 2.3 and 4.5 h for the fully and partially immersed mites (Table 1).

## Discussion

Human sebum, which consists mainly of triglycerides and fatty acids (57.5%) as well as wax esters (26%), squalene (12%), and cholesterol (4.5%), is an optimal living environment for *D. folliculorum* and *D. brevis* [15, 16]. These compounds protect the mites against water loss and are a source of food. Hormones increasing sebum production may also affect the course of demodicosis and increase the possibility of infection. Demodicosis and seborrheic dermatitis have been associated with polycystic ovary syndrome (PCOS), probably due to the increased discharge of sebum promoted by androgens. *D. folliculorum* has been found to be more predominant in women with PCOS [17]. An important epidemiological task is to determine whether and how long these permanent human parasites can survive without a host. Our previous investigations demonstrated that *Demodex* spp. immersed in saline survived on average for 82 h [18]. In the present study, the mites in the control group survived for 41 h, which was shorter than in physiological saline. The shortest viability time was recorded in the powder cream substrate. The survival time in the mascara was longer, and the lipstick substrate ensured the longest viability of the mites. The analysis of the *Demodex* survival in the cosmetics was focused on the first six chemical components that may potentially affect the viability of these mites. Demodicidal activity has been shown for many compounds, i.e., 100% tea tree oil (TTO), 100% caraway oil, 99% ethanol, 10% povidone-iodine, 4% pilocarpine, and 4% terpinen-4-ol (T4O), but only some of them can be used as cosmetic ingredients due to their intrinsic toxicity and the risk of irritation of eyes and skin [19–22].

The powder used in the study contained the following ingredients from the top of the list: dimethicone, water, dipropylene glycol, alcohol denat., nylon-12, and butylene glycol. Dimethicone and denatured alcohol seem to be the most important components influencing *Demodex* survival. Dimethicone has a protective effect, as it protects the skin against external factors. It forms a film on the skin producing a feeling of smoothness, softens the skin, and nourishes the hair [23]. The compound is used not only as a cosmetic ingredient but also as an agent against head lice *Pediculus humanus capitis* [24]. It enters the respiratory system of lice and causes their death within 0.5 h [25]. Other authors suggest that the death of lice does not occur via hypoxia but via inhibition of water excretion [26]. The average survival time of *Demodex* mites that were fully immersed in the powder cream was comparable to that of dimethicone-treated head lice (0.78 h). Mites that were partially immersed in the powder cream survived over twice as long (2.16 h). This may indicate that dimethicone had the most significant effect on the survival of *Demodex* in this substrate.

Another component, i.e., dipropylene glycol (DPG), is used e.g. as a solvent in cosmetics and personal care products [27]. There are no data on the effect of this substance on mites. Alcohol denat. is a general term used by the cosmetics industry to refer to denatured alcohol, which is added to a wide range of products [28]. It acts as an effective preservative, prevents spoilage of cosmetics, and serves as an antibacterial agent [29]. In 75% and 100% ethyl alcohol, *Demodex* can survive for approx. 150 and 4 min, respectively [20]. The concentration of the alcohol in the powder cream is definitely lower; however, its presence may be associated with the shorter survival time of the mites in this substrate. Since it is usually applied to the face with fingers, powder cream can be a source of *Demodex* infections when used by several persons within a short time (tens of minutes). It seems that the dimethicone ingredient is crucial for *Demodex* survival in this cosmetic.

The *Demodex* mites were immersed in the mascara partially, as its dark colour would have prevented observation at full immersion. The small size of these mites impeded observations of the movement of legs, which was the basic survival criterion. The partially immersed specimens were found to survive in the mascara for 21 h on average.

The first main components of the mascara are water, *Copernicia* cerifera cera, cera alba, glyceryl stearate, *Euphorbia* cerifera cera, and stearic acid. Water provides *Demodex* mites with the required humidity; hence, this substance does not seem to have a negative effect on the survival of these parasites. Another ingredient, i.e., *Copernicia* cerifera cera, is wax extracted from leaves and buds of the Brazilian palm *Copernicia prunifera*. This substance is considered hypoallergenic, although allergic contact dermatitis has been reported in the literature [30]. In turn, the cera alba beeswax forms an occlusive layer on the surface of the skin or mites, preventing excessive evaporation of water. In humans, cera alba may be a causative agent of allergic reactions [31]. *Euphorbia* cerifera cera is a wax component as well; yet, the effect of waxes on *Demodex* mites is unknown at present [32].

Glyceryl stearate is used in cosmetic preparations as an emollient, emulsifier, and stabiliser. This component was found to be slightly toxic in acute oral toxicity studies in rats [33]. However, as in the case of waxes, its effect on *Demodex* has not been described.

*Demodex* mites immersed in the lipstick substrate had the longest survival time (fully immersed: 38.5 h, partially immersed: 148.1 h). The main components of this product include various types of emollients preventing water evaporation. Cera microcristallina (crystalline mineral wax), i.e. the so-called greasy emollient, is the first ingredient on the list. It is used in skin and hair care formulations to form an occlusive layer (film) on their surface. Thus, it prevents excessive evaporation of water. Similar effects are provided by the other ingredients, i.e., octyldodecanol, hydrogenated polydecene, *Ricinus communis* seed oil, and cetyl palmitate [34–37]. In turn, ethylhexyl methoxycinnamate is one of the most commonly used UVB filters in sunscreen products [38]. There are no data about its acaricidal activity.

Most compounds contained in the lipstick have lubricating properties. Given the long survival of the *Demodex* mites in this cosmetic, it can be concluded that the parasites find such an environment favourable as they feed on lipids [39]. Transmission via shared lipstick may take place mainly in the case of perioral demodicosis.

The present study was conducted to determine whether widely available cosmetics, e.g., testers in drugstores or cosmetics used by makeup artists, could be a potential source of *Demodex* infection. The study was focused on facial cosmetics, as these parasites most frequently colonise the face. The high prevalence of *Demodex* spp. in humans indicates widespread occurrence of these mites, whereas the routes of their spread have not been fully elucidated.

Our results indicate that mites can be spread in shared cosmetics. Shared facial powder is associated with the likelihood of transferring *Demodex* spp. when the cosmetic is used by several persons within a short time (average survival time: 47 min). Shared lipsticks may promote *Demodex* spp. infection (average survival time: 38.5 h), although the number of mites on the lips is inconsiderable. The analysed mites die faster in powder, but there is a greater risk of transmission thereof from the face onto the cosmetic with user's fingers. A high risk of transmission of *Demodex* spp. is associated with using the same mascara, in which the mite was found to survive up to 56 h.

The survival of *Demodex* spp. in cosmetics is undoubtedly influenced by their chemical composition as well as the mode of use. Since testers in drugstores can be opened and applied several times on the face with fingers instead of disposable spatulas, there is a risk that mites present on the face will be transferred to another person using the same tester. Testing mascara poses a high risk, as *Demodex* mites are typically present on eyelashes. The next person using the same brush can be infected in this way.

Based on information provided by drugstore staff and our observations, we estimate that from a dozen to even several tens of persons per day may use makeup testers. Some of them test such cosmetics as powder on the hand, but a large group uses cosmetic testers for partial or full makeup. Similarly, customers check mascara testers on their eyelashes. Such observations of consumer behaviour have supported our thesis that investigation of the survival of *Demodex* mites in cosmetics is an epidemiologically important issue.

## Conclusions

Facial cosmetics shared at a short interval may contribute to *Demodex* transfer between the users. Therefore, cosmetics available to many customers should be tested with the use of disposable spatulas and makeup cosmetics should only serve for personal use. Furthermore, addition of not only bactericidal but also demodicidal compounds to makeup cosmetic formulas should be considered.

## Supplementary Information

Below is the link to the electronic supplementary material.

Supplementary file1 (PDF 1894 KB)

## References

[1] Aylesworth R, Vance JC (1982). *Demodex folliculorum* and *Demodex brevis* in cutaneous biopsies. J Am Acad Dermatol.

[2] Yokoyama T, Yamaguchi R, Itoh T, Toh U, Nakagawa S, Kage M (2014). Detection of *Demodex folliculorum* from nipple discharge. Diagn Cytopathol.

[3] Sędzikowska A, Osęka M, Grytner-Zięcina B (2016). Ocular symptoms reported by patients infested with *Demodex* mites. Acta Parasitol.

[4] Huang Y, He H, Sheha H, Tseng SC (2013). Ocular demodicosis as a risk factor of pterygium recurrence. Ophthalmology.

[5] Hsu CK, Hsu MM, Lee JY (2009). Demodicosis: a clinicopathological study. J Am Acad Dermatol.

[6] Liu J, Sheha H, Tseng SC (2010). Pathogenic role of *Demodex* mites in blepharitis. Curr Opin Allergy Clin Immunol.

[7] Tarkowski W, Owczyńska M, Błaszczyk-Tyszka A, Młocicki D (2015). *Demodex* mites as potential etiological factor in chalazion - a study in Poland. Acta Parasitol.

[8] Moran EM, Foley R, Powell FC (2017). *Demodex* and rosacea revisited. Clin Dermatol.

[9] Douglas A, Zaenglein AL (2019). A case series of demodicosis in children. Pediatr Dermatol.

[10] Trager MH, Queen D, Chen D, Hodak E, Geskin LJ (2020). *Demodex*-induced follicular mucinosis of the head and neck mimicking folliculotropic mycosis fungoides. JAAD Case Rep.

[11] Przydatek-Tyrajska R, Sędzikowska A, Bartosik K (2020). Primary facial demodicosis as a health problem and aesthetic challenge: a case report. J Cosmet Dermatol.

[12] Palopoli MF, Fergus DJ, Minot S, Pei DT, Simison WB, Fernandez-Silva I, Thoemmes MS, Dunn RR, Trautwein M (2015). Global divergence of the human follicle mite *Demodex folliculorum*: persistent associations between host ancestry and mite lineages. Proc Natl Acad Sci USA.

[13] Czepita D, Kuźna-Grygiel W, Czepita M, Grobelny A (2007). *Demodex folliculorum* and *Demodex brevis* as a cause of chronic marginal blepharitis. Ann Acad Med Stetin.

[14] Rusiecka-Ziółkowska J, Nokiel M, Fleischer M (2014). *Demodex* - an old pathogen or a new one? Adv. Clin Exp Med.

[15] Greene RS, Downing DT, Pochi PE, Strauss JS (1970). Anatomical variation in the amount and composition of human skin surface lipid. J Invest Dermatol.

[16] Picardo M, Ottaviani M, Camera E, Mastrofrancesco A (2009). Sebaceous gland lipids. Dermatoendocrinol.

[17] Benk Silfeler D, Keskin Kurt R, Kaya OA, Yengil E, Hamamci B, Okyay AG, Beyazit A (2015). *Demodex folliculorum* in polycystic ovary syndrome patients. Eur Rev Med Pharmacol Sci.

[18] Sędzikowska A, Osęka M, Grytner-Zięcina B, Roman B, Jaremko E (2014). Effect of metronidazol, mercury oxide and essentials oils on the in vitro survivability of *Demodex* mites. Okulistyka.

[19] Norn MS (1970). *Demodex folliculorum*. Incidence and possible pathogenic role in the human eyelid. Acta Ophthalmol.

[20] Gao YY, Di Pascuale MA, Li W, Baradaran-Rafii A, Elizondo A, Kuo CL, Raju VK, Tseng SC (2005). In vitro and in vivo killing of ocular *Demodex* by tea tree oil. Br J Ophthalmol.

[21] Tighe S, Gao YY, Tseng SC (2013). Terpinen-4-ol is the most active ingredient of tea tree oil to kill *Demodex* mites. Transl Vis Sci Technol.

[22] Kabat AG (2019). In vitro demodicidal activity of commercial lid hygiene products. Cli Ophthalmol.

[23] Becker LC, Bergfeld WF, Belsito DV, Hill RA, Klaassen CD, Liebler DC, Marks JG, Shank RC, Slaga TJ, Snyder PW, Andersen FA (2014). Safety assessment of dimethicone crosspolymers as used in cosmetics. Int J Toxicol.

[24] Ihde ES, Boscamp JR, Loh JM, Rosen L (2015). Safety and efficacy of a 100% dimethicone pediculocide in school-age children. BMC Pediatr.

[25] Candy K, Brun S, Nicolas P, Durand R, Charrel RN, Izri A (2018). Do drowning and anoxia kill head lice?. Parasite.

[26] Burgess IF (2009). The mode of action of dimeticone 4% lotion against head lice, Pediculus capitis. BMC Pharmacol.

[27] Fowles JR, Banton MI, Pottenger LH (2013). A toxicological review of the propylene glycols. Crit Rev Toxicol.

[28] Anderson FA (2008). Final report of the safety assessment of Alcohol Denat., including SD Alcohol 3-A, SD Alcohol 30, SD Alcohol 39, SD Alcohol 39-B, SD Alcohol 39-C, SD Alcohol 40, SD Alcohol 40-B, and SD Alcohol 40-C, and the denaturants, Quassin, Brucine Sulfate/Brucine, and Denatonium Benzoate. Int J Toxicol.

[29] Halla N, Fernandes IP, Heleno SA, Costa P, Boucherit-Otmani Z, Boucherit K, Rodrigues AE, Ferreira ICFR, Barreiro MF (2018). Cosmetics preservation: a review on present strategies. Molecules.

[30] Alrowaishdi F, Colomb S, Guillot B, Raison-Peyron N (2013). Allergic contact cheilitis caused by carnauba wax in a lip balm. Contact Dermatit.

[31] Jensen CD, Andersen KE (2006). Allergic contact dermatitis from cera alba (purified propolis) in a lip balm and candy. Contact Dermatit.

[32] Moore AF (1984). Final report on the safety assessment of candelilla wax, carnauba wax, japan wax, and beeswax. J Am Coll Toxicol.

[33] Fisher K (1982). Final report on the safety assessment of glyceryl stearate and glyceryl stearate/SE. J Am Coll Toxicol.

[34] Williams SD, Schmitt WH (1992). Chemistry and technology of the cosmetics and toiletries industry.

[35] Monti M, Motta S (2004). Polydecene oligomers versus mineral oils: the rationale for use in dermatological preparations. JAAD.

[36] Johnson W (2007). Final report on the safety assessment of *Ricinus communis* (Castor) seed oil, hydrogenated castor oil, glyceryl ricinoleate, glyceryl ricinoleate SE, ricinoleic acid, potassium ricinoleate, sodium ricinoleate, zinc ricinoleate, cetyl ricinoleate, ethyl ricinoleate, glycol ricinoleate, isopropyl ricinoleate, methyl ricinoleate, and octyldodecyl ricinoleate. Int J Toxicol.

[37] Williams SD, Schmitt WH (1996). Chemistry and technology of the cosmetics and toiletries industry.

[38] Durand L, Habran N, Henschel V, Amighi K (2010). Encapsulation of ethylhexyl methoxycinnamate, a light-sensitive UV filter, in lipid nanoparticles. J Microencapsul.

[39] Desch C, Nutting WB (1972). *Demodex folliculorum* (Simons) and *D. brevis* Akbulatova of man: redescription and reevaluation. J Parasitol.

