# Supplementary figures and images for: Shared Makeup Cosmetics as a Route of Demodex folliculorum Infections
Source: Acta Parasitol. 2021 Jan 19;66(2):631–7. doi: 10.1007/s11686-020-00332-w (PMC8166727; doi:10.1007/s11686-020-00332-w)

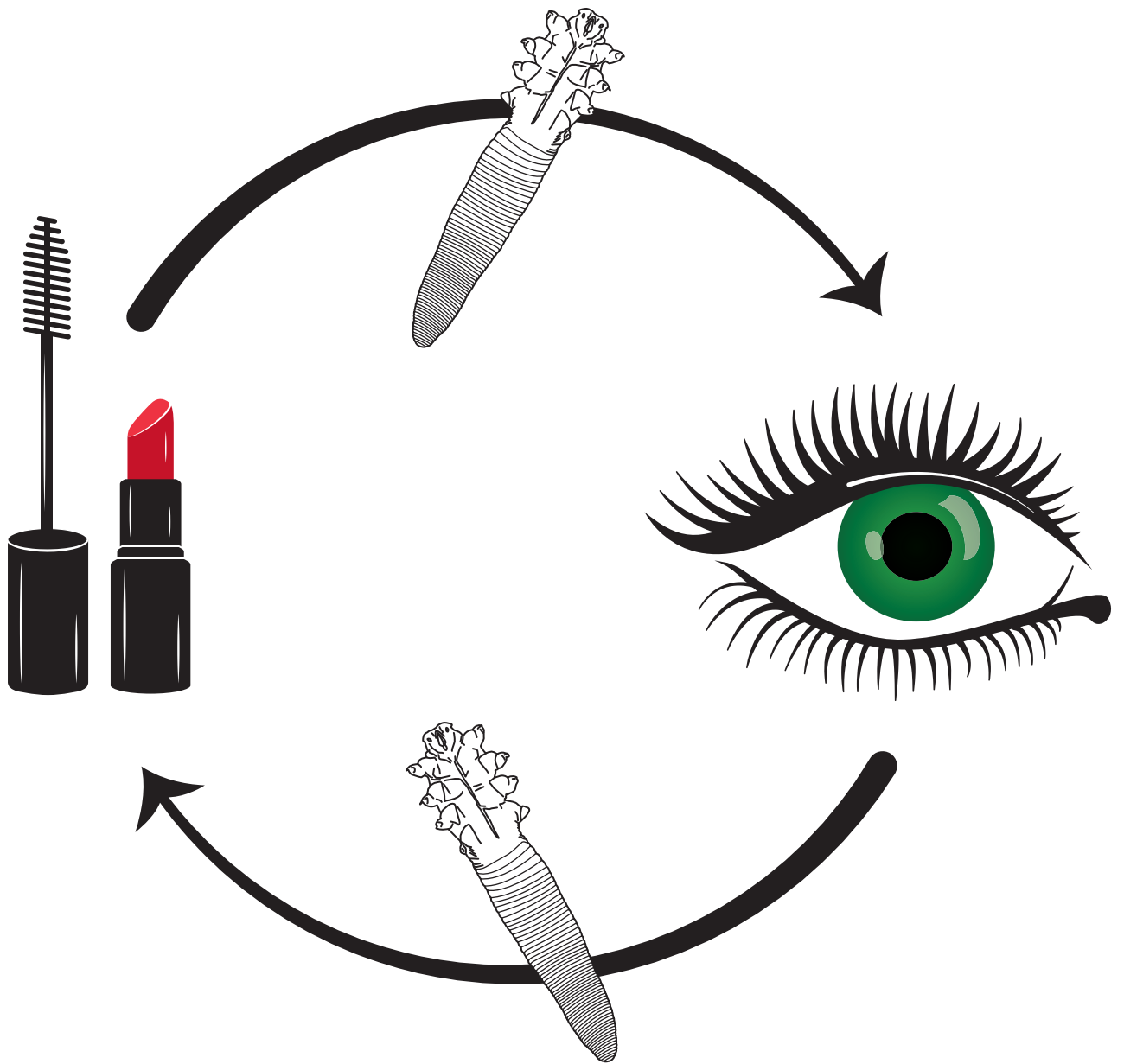

Supplement: Supplementary file 1 — Supplementary file1 (PDF 1894 KB) [file 11686_2020_332_MOESM1_ESM.pdf]
